# Increased miR‐223 expression in foetal organs is a signature of acute chorioamnionitis with systemic consequences

**DOI:** 10.1111/jcmm.13377

**Published:** 2017-10-30

**Authors:** JoonHo Lee, Chong Jai Kim, Jung‐Sun Kim, Deug‐Chan Lee, Sejin Ahn, Bo Hyun Yoon

**Affiliations:** ^1^ Department of Obstetrics and Gynecology Institute of Women's Life Medical Science Yonsei University College of Medicine Yonsei University Health system Seoul Korea; ^2^ Department of Pathology University of Ulsan College of Medicine Seoul Korea; ^3^ Department of Pathology Sungkyunkwan University School of Medicine Seoul Korea; ^4^ Department of Biomedical Technology Kangwon National University College of Biomedical Science Chuncheon Korea; ^5^ Department of Obstetrics and Gynecology Seoul National University College of Medicine Seoul Korea

**Keywords:** acute chorioamnionitis, autopsy, FoxO1, foetus, miR‐223

## Abstract

Acute chorioamnionitis, frequently observed in preterm placentas, is a major risk factor for the development of infection and non‐infection‐related adverse perinatal outcomes. MicroRNAs play important roles in immune cell development and function as well as in the development of cancers and neurologic diseases. We sought to investigate the changes in microRNA‐223 (miR‐223) expression and the functional significance of the changes in miR‐223 expression in foetal organs in the presence of acute chorioamnionitis. Using formalin‐fixed, paraffin‐embedded (FFPE) tissue samples from foetal or neonatal autopsy cases, which are the most practical option to study the changes in several organs simultaneously, miR‐223 expression profiles in foetal thymus, lung and liver were compared between cases with and without acute chorioamnionitis. Total RNA was extracted from FFPE specimens and qRT‐PCR was conducted. miR‐223‐3p expression levels in foetal thymus (2.55‐fold), lung (1.93‐fold) and liver (1.70‐fold) were significantly higher in cases with acute chorioamnionitis than in those without. Transfection of pre‐miR‐223‐3p in Jurkat cells and luciferase assay and ribonucleoprotein immunoprecipitation followed by qRT‐PCR analysis confirmed the binding of miR‐223 to the 3′ untranslated region (3′UTR) of forkhead box O1 (FoxO1) mRNA and the regulation of FoxO1 by miR‐223. We report for the first time that foetuses with inflammation in the chorioamniotic membranes show increased expression of miR‐223 in the thymus, lung and liver. Furthermore, FoxO1 is a target of miR‐223. These findings suggest that post‐transcriptional regulation of genes by miR‐223 is a component of the foetal inflammatory response, which has systemic consequences in the foetus.

## Introduction

Acute chorioamnionitis is frequently diagnosed in preterm placentas and is a major risk factor for the development of infection and non‐infection‐related perinatal morbidity and mortality 1. The presence of acute chorioamnionitis indicates the acute inflammatory responses of mothers and foetuses to bacterial infection in the gestational sac 2 and is generally considered as a histologic hallmark for intra‐amniotic infection and/or inflammation 3, 4, given that the elevation of many intra‐amniotic inflammatory markers has been reported to be associated with acute chorioamnionitis 1.

MicroRNAs (miRNAs) are small, single‐stranded, non‐coding RNA (usually 18–24 nucleotides) 5 that play an important role in the post‐transcriptional regulation of gene expression. Approximately 2,000 miRNAs have been reported, and about 60% of protein synthesis genes are thought to be regulated by miRNAs 6, 7, 8, 9. Aberrations of miRNAs expression are associated with the development of major human diseases such as cancers and neurologic diseases 10. As miRNAs are relatively stable, they have been successfully investigated using formalin‐fixed, paraffin‐embedded (FFPE) tissue samples 11, 12, 13, 14, 15, 16. In the obstetric fields, however, the number of studies on miRNAs in placental tissues, trophoblasts and maternal blood samples is limited in cases with congenital anomalies and perinatal diseases 17, 18, 19, 20, 21, 22, 23, 24, 25, 26, 27.

Recently, Montenegro *et al*. have reported the differential expression of subsets of miRNAs with the progression of gestation and inflammation in human chorioamniotic membranes 24. Considering the fact that the placenta is a foetal organ and foetal exposure to intra‐amniotic infection/inflammation is systemic in nature, we postulated that the differential expression of miRNAs confirmed in the placenta could be shared by various foetal organs along with systemic biological effects in the foetus. As the differential expression of miRNA‐223 (miR‐223) in the placenta was found according to the presence or absence of acute chorioamnionitis in the previous study 24, the current study was conducted to determine whether there is a differential expression of miR‐223 in foetal organs according to the presence or absence of acute chorioamnionitis and also whether it has potentially functional systemic consequences in the foetus. Foetal or neonatal autopsy materials were used for the analysis, and we further identified a functional target of miR‐223.

## Materials and methods

### Study design

Autopsy cases of foetuses and neonates met the following criteria: (*i*) cases of intrauterine foetal death (IUFD) or neonatal death whose autopsies were performed at Seoul National University Hospital with written informed consent for autopsy and (*ii*) cases whose tissue amount collected and preserved during autopsy were adequate for this study. All FFPE tissue blocks of various foetal organs from autopsy cases were preserved. Cases with acute chorioamnionitis in the placenta were compared to those without acute chorioamnionitis in the placenta and with appropriate‐for‐gestational age (AGA) foetuses or neonates. Acute chorioamnionitis was diagnosed when neutrophil infiltration was found in the extra‐placental chorioamniotic membranes or chorionic plate of the placenta, or in the umbilical vessel walls or Wharton's jelly, using previously published criteria 2. A review of the mothers’ and babies’ medical records and autopsy reports was performed for the confirmation of final diagnosis of autopsy, as well as for the data collection of the pathological results of gross specimen and microscopic examination, respectively. The collection and use of materials for research purposes were approved by the Institutional Review Board of Seoul National University Hospital, Seoul, Republic of Korea.

### RNA isolation

Formalin‐fixed, paraffin‐embedded (FFPE) tissue blocks were cut into 5‐μm‐thick slices, and paraffin was removed from FFPE tissue slices with xylene. After washing with ethanol, tissue samples were lysed with Proteinase K. Extraction of total RNA from tissue lysates was performed with a High Pure RNA Paraffin Kit (Roche Applied Science, Indianapolis, IN, USA). Samples were treated with DNase and Proteinase K to completely remove DNA and protein. Extracted RNA samples were stored at −80°C until analysis.

### Real‐time quantitative reverse transcription–polymerase chain reaction (qRT‐PCR)

Reverse transcription was conducted using the TaqMan MicroRNA Reverse Transcription Kit (Applied Biosystems, Foster City, CA, USA) for miRNA analysis and the Improm‐II Reverse Transcription System (Promega, Madison, WI, USA) for mRNA analysis. All PCR analyses were carried out using TaqMan Assays (Applied Biosystems). RPLP0 (Hs02992885_s1) was used for normalization of FoxO1 mRNA expression (Hs01054576_m1), and a custom‐designed TaqMan assay for 5S ribosomal RNA (4332078) was used for normalization of miR‐223 expression (hsa‐miR‐223‐3p, 002295) 20, 24, 28. Reactions were carried out using the 7500 Fast Real‐Time PCR System (Applied Biosystems). For the ribonucleoprotein immunoprecipitation (RNP‐IP) experiment, the RNA isolated from RNP‐IP was reverse‐transcribed to cDNA using ReverTra Ace qPCR RT Master Mix (TOYOBO, Osaka, Japan). The relative gene expression was determined with THUNDERBIRD SYBR qPCR Mix (TOYOBO), and cDNA amplified by oligo dA (15 mer) was used for normalization. The primer pairs were synthesized by Macrogen (Seoul, Republic of Korea) as follows: FoxO1 (forward: 5′‐GCCTGTAGCAACCTAAACTG‐3′ and reverse: 5′‐GGGCTTTCCACATGACTTGA‐3′) and Oligo dA (5′‐AAAAAAAAAAAAAAA‐3′). The qRT‐PCR was performed using a StepOne Plus Real‐Time PCR system (Applied Biosystems). The PCR products were resolved on 3% agarose gel, stained with ethidium bromide, and analysed using Omega Lum G (Aplegen Inc., Pleasanton, CA, USA).

### Cell culture

Jurkat cells (human T lymphocyte cell line) were incubated in RPMI 1640 medium (Hyclone, Logan, UT, USA) supplemented with 10% heat‐inactivated foetal bovine serum (Hyclone) and a 1% solution containing penicillin and streptomycin (Hyclone) in a humidified atmosphere of 5% CO2 at 37°C.

### Transfection of Jurkat cells with miR‐223 mimic

Pre‐miR‐223‐3p molecules (PM12301, Ambion Inc., Foster City, CA, USA) were used to add the function of exogenous miR‐223 to Jurkat cells (human T lymphocyte cell line). For the transfection with pre‐miR‐223‐3p, 1 × 10^6^ Jurkat cells were split in six‐well plates, kept overnight and transfected with pre‐miR‐223‐3p at the concentration of 50 nM, using siPORT™ *Neo*FX™ Transfection Agent (Ambion Inc.) and optiMEM (Invitrogen, Carlsbad, CA, USA). Control cells were transfected with miRNA precursor molecules‐negative controls (scramble) (Ambion Inc.) at equimolar concentrations. The cells were harvested 48 hrs after transfection for the isolation of total RNA and protein. Additionally, to assess the effects of miR‐223 binding to the 3′ untranslated region (3′ UTR) of FoxO1 mRNA in Jurkat cells with the RNP‐IP experiment, 150 pmole of pre‐miR‐223‐3p (MC12301, Ambion Inc.) was transfected to Jurkat cells using the Neon transfection system (Invitrogen) at 1,325 mV with three 10‐ms pulses. Control cells were transfected with mirVana™ miRNA mimic‐negative control #1 (4464058, Ambion Inc) at equimolar concentrations. Jurkat cells (1 × 10^7^) transfected with pre‐miR‐223 or the negative control molecule were harvested after 48 hrs for RNP‐IP.

### Generation of FoxO1 3′ UTR reporter construct

The FoxO1 3′ UTR carrying a putative miR‐223 binding site was PCR‐amplified, sequence‐verified and cloned into a *Spe* I and *Hind* III site of the pMIR‐REPORT™ miRNA Expression Reporter Vector (Ambion Inc.). PCR was carried out using an upstream primer (5′‐ATAATACTAGTCAGATGGGTAGCAAATGGAATAGAACTTAC‐3′) bearing a *Spe* I site and a downstream primer (5′‐ATAATAAGCTTTTAGATCCTTCTCAAGAACACAAGAGGAAC‐3′) bearing a *Hind* III site. The 325‐bp PCR product was purified in a 1% agarose gel after electrophoresis with a PureLink™ Quick Gel Extraction Kit (Invitrogen), and its sequence was confirmed by DNA sequencing using an ABI 3100 sequencer (Applied Biosystems).

### Reporter gene assay

To assess miR‐223 repressing the 3′ UTR of FoxO1 mRNA in Jurkat cells, 1.5 × 10^6^ cells were transfected with 200 ng of pMIR‐REPORT™ Reporter Vector (Ambion Inc.) or 200 ng of pMIR‐REPORT‐FoxO1‐3′ UTR, 10 ng of Renilla luciferase reporter pSV40‐RL (transfection control; Promega) and 50 nM of miR‐223‐3p precursor molecules or equal amounts of miRNA precursor molecules‐negative controls. At 48 hrs after transfection, luciferase assays were performed using the Dual‐Luciferase Reporter Assay System (Promega) according to the manufacturer's instructions. All experiments were carried out five times.

### Ribonucleoprotein immunoprecipitation (RNP‐IP)

Transfected Jurkat cells were collected by centrifugation and then washed twice with cold nuclease‐free PBS. The cell pellet was lysed with lysis buffer (100 mM KCl, 5 mM MgCl_2_, 10 mM Tris, 0.5% NP40) supplemented with 1 mM β‐mercaptoethanol, 100 U/ml Protector RNase inhibitor (Roche), Halt protease and phosphatase inhibitor (Thermo scientific, Rockford, IL, USA) and 25 μM MG 132 (Sigma‐Aldrich, St. Louis, MO, USA). The lysate was incubated in ice for 30 min., and the supernatant was collected by centrifugation at 15,000 × *g* for 5 min. at 4°C. The supernatant was pre‐cleared with 20 μl of Protein G Plus agarose beads (Santa Cruz Biotechnology Inc., Santa Cruz, CA, USA) in a rotating wheel for 30 min. at 4°C. As the control for the comparison of extracted protein among samples, 5% of cleared lysate was used. After pre‐clearing, 1 μg of AGO2 (MABE253, Millipore, Darmstadt, Germany) or normal rat IgG (sc‐2026, Santa Cruz Biotechnology Inc.) antibody was incubated with the pre‐cleared lysate overnight at 4°C. Twenty microlitres of Protein G Plus agarose beads (Santa Cruz Biotechnology Inc.) was incubated with the mixture for 1 hr at 4°C. The beads were washed four times with cold NT2 buffer supplemented with 1 mM β‐mercaptoethanol and 20 mM EDTA. As the control for comparison of the precipitated antibody among samples, 5% of the immunoprecipitated pellet was used. After the last washing, the immunoprecipitated pellet was incubated in NT2 buffer supplemented with 100 μg/ml Proteinase K (Sigma‐Aldrich) for 30 min. at 55°C. For the isolation of RNA, Isol‐RNA Lysis reagent (5 prime) was added directly to the immunoprecipitated pellet. The RNA was precipitated with isopropanol, and the pellet was washed with 70% ethanol. The washed pellet was then treated with DNase I (Ambion Inc.) according to manufacturer's instruction.

### Immunoblotting

Total proteins were isolated from Jurkat cells using RIPA buffer (Sigma‐Aldrich, St. Louis, MO, USA) containing a protease inhibitor cocktail (Roche). Ten to twenty microlitres of protein was subjected to 10% SDS–polyacrylamide gel electrophoresis and electrotransferred onto nitrocellulose membranes. The membranes were blocked with 5% non‐fat dry milk in Tris‐buffered saline containing 0.1% v/v Tween‐20 and were incubated overnight at 4°C with a rabbit polyclonal anti‐FoxO1 (1:1,000; Cell Signaling, Danvers, MA, USA) and a mouse monoclonal anti‐β‐actin (1:5,000; Sigma‐Aldrich). A horseradish peroxidase‐conjugated anti‐rabbit or anti‐mouse IgG was used as a secondary antibody. Signals were detected by chemiluminescence using the ChemiGlow West kit (Alpha Innotech, San Leandro, CA, USA). Densitometric analyses were performed using the Multi Gauge software version 3.1 of the imaging system LAS‐4000 (Fujifilm, Tokyo, Japan). For the RNP‐IP experiment, the controls of RNP‐IP were confirmed by immunoblotting analysis probed with anti β‐actin and anti‐rat‐IgG antibody. After preparation of controls during RNP‐IP (as mentioned above), the samples were boiled for 10 min., electrophoresed by 10% sodium dodecyl sulphate–polyacrylamide gel electrophoresis (SDS‐PAGE), and electrotransferred onto polyvinylidene difluoride membranes (Amersham Hybomd‐P; GE Healthcare, Buckinghamshire, UK). Membranes were blocked with 1% skim milk in TBS containing 0.05% Tween‐20 for 1 hr. The membrane of the control for comparison of the extracted protein among the samples was incubated with a mouse monoclonal anti‐β‐actin antibody (1:5,000; ab8226, Abcam, Cambridge, UK) for 1 hr. After three washes in TBS containing 0.05% Tween‐20, the membranes were incubated with horseradish peroxidase linked secondary antibodies (1:5,000; sc‐2005, Santa Cruz Biotechnology Inc.) for 1 hr. The membrane of the control for comparison of the precipitated antibody among the samples was incubated with horseradish peroxidase linked secondary antibodies (sc‐2006, Santa Cruz Biotechnology Inc.) for 1 hr. After three washes in TBS containing 0.05% Tween‐20, the chemiluminescent signals of both membranes were detected using WesternBright Peroxide chemiluminescent detection reagent (Advansta, Menlo Park, CA, USA).

### Immunohistochemistry

Immunohistochemistry was performed to assess FoxO1 protein expression in foetal thymus, lung and liver tissues. Five‐micrometre‐thick sections of FFPE blocks of foetal thymus, lung and liver tissues from autopsy cases were placed on silanized slides. For antigen retrieval, the sections were heated in citrate buffer (pH 6.0) for 30 min. using a microwave. Then, endogenous peroxidases were quenched with DAKO blocking reagent (Dako Corp., Carpinteria, CA, USA) for 15 min. The sections were incubated with a rabbit monoclonal anti‐FOXO1 antibody (1:100, Cell Signaling) and subsequently incubated with a biotinylated anti‐immunoglobulin and streptavidin‐peroxidase (DAKO LSAB kit, Dako Corp.). Rabbit mAb IgG isotype (1:100; Cell Signaling) was used as a negative control. Colour was developed using 3, 3‐diaminobenzidine tetrahydrochloride (DAB) for 3 min., and then, the sections were counterstained with haematoxylin for 1 min.

### Statistical analysis

To compare the continuous variables, Student's *t*‐test or Mann–Whitney *U*‐test for the independent variables and the paired *t*‐test or Wilcoxon signed‐rank test for the related variables were performed as appropriate. For the categorical variables, Fisher's exact test or chi‐square test was conducted as appropriate. All statistical analyses were performed using SPSS version 19.0 (SPSS Inc., Chicago, IL, USA), and a *P*‐value of <0.05 was considered significant.

## Results

### miR‐223 expression in various foetal organs

**Figure 1:**
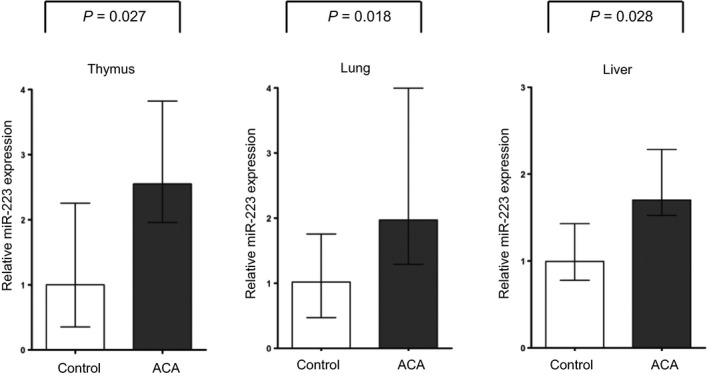
miR‐223 expression in foetal organs. miR‐223‐3p expression levels in the foetal thymus (2.55‐fold), lung (1.93‐fold) and liver (1.70‐fold) were higher in cases with acute chorioamnionitis than in those without the lesion (*P *<* *0.05 for each). ACA, acute chorioamnionitis. Relative expressions are presented as columns (median) and error bars (interquartile range).

Eighteen FFPE samples from autopsy cases for thymus (*N *=* *5 for control *versus N *=* *13 for acute chorioamnionitis), 26 samples for lung (*N *=* *10 for control *versus N *=* *16 for acute chorioamnionitis) and 11 samples for liver (*N *=* *5 for control *versus N *=* *6 for acute chorioamnionitis) were available for the analysis of miR‐223 expression. Demographic findings and clinical information in this study are reported in Table 1. miR‐223‐3p expression levels in the foetal thymus (2.55‐fold), lung (1.93‐fold) and liver (1.70‐fold) were higher in cases with acute chorioamnionitis than in those without the lesion (*P *<* *0.05 for each, Fig. 1).

**Table 1:** Demographics of study population

Characteristics	Acute chorioamnionitis		
	No (*N *=* *5)	Yes (*N *=* *13)^b^	
Thymus			
Maternal age (yr)^a^	29 (25–42)	32 (25–43)	0.458
Nulliparity (%)	40.0	25.0	0.600
Gestational age at delivery (week)^a^	21.3 (19.3–24.0)	22.7 (18.0–26.7)	0.554
Birthweight (g)^a^	370 (185–690)	560 (120–820)	0.343
Foetal gender (male, %)	100.0	54.5	0.231
Indication of autopsy			>0.999
Spontaneous abortion/preterm delivery (%)	80.0	76.9	
Cervical insufficiency (incompetence os of cervix)	20.0	23.1	
Preterm premature rupture of membranes	20.0	30.8	
Preterm labour	40.0	23.1	
Induced/Therapeutic abortion/preterm delivery (%)	20.0	23.1	
Oligohydramnios	20.0	0.0	
Placental abruption	0.0	7.7	
Therapeutic abortion	0.0	7.7	
Major structural abnormalities	0.0	7.7	

Characteristics	Acute chorioamnionitis		
	No (*N *=* *5)	Yes (*N *=* *6)	
Liver			
Maternal age (yr)^a^	36 (29–42)	32 (29–43)	0.313
Nulliparity (%)	40.0	0.0	0.182
Gestational age at delivery (week)^a^	21.3 (18.0–24.0)	23.5 (21.4–28.3)	0.082
Birthweight (g)^a^	370 (130–690)	585 (120–820)	0.361
Foetal gender (male, %)	80.0	60.0	>0.999
Indication of autopsy			0.455
Spontaneous abortion/preterm delivery (%)	100.0	66.7	
Cervical insufficiency (Incompetence os of cervix)	20.0	16.7	
Preterm premature rupture of membranes	40.0	50.0	
Preterm labour	40.0	0.0	
Induced/Therapeutic abortion/preterm delivery (%)	0.0	33.3	
Oligohydramnios	0.0	0.0	
Placental abruption	0.0	16.7	
Therapeutic abortion	0.0	0.0	
Major structural abnormalities	0.0	16.7	

Characteristics	Acute chorioamnionitis		
	No (*N *=* *10)	Yes (*N *=* *16)	
Lung			
Maternal age (yr)^a^	29 (21–42)	32 (25–43)	0.510
Nulliparity (%)	44.4	28.6	0.657
Gestational age at delivery (week)^a^	20.5 (16.0–24.0)	22.4 (16.0–24.7)	0.356
Birthweight (g)^a^	350 (130–690)	530 (120–820)	0.233
Foetal gender (male, %)	70.0	46.7	0.414
Indication of autopsy			0.340
Spontaneous abortion/preterm delivery (%)	70.0	87.5	
Cervical insufficiency (Incompetence os of cervix)	30.0	18.8	
Preterm premature rupture of membranes	20.0	50.0	
Preterm labour	20.0	18.8	
Induced/Therapeutic abortion/preterm delivery (%)	30.0	12.5	
Oligohydramnios	10.0	0.0	
Placental abruption	0.0	6.8	
Therapeutic abortion	10.0	0.0	
Major structural abnormalities	10.0	6.8	

a Median (range).

b Two cases were excluded in the immunohistochemical analysis of FoxO1 protein expression in foetal thymus.

### Forkhead box O1 (FoxO1) as a putative target of miR‐223 in Jurkat T cells

**Figure 2:**
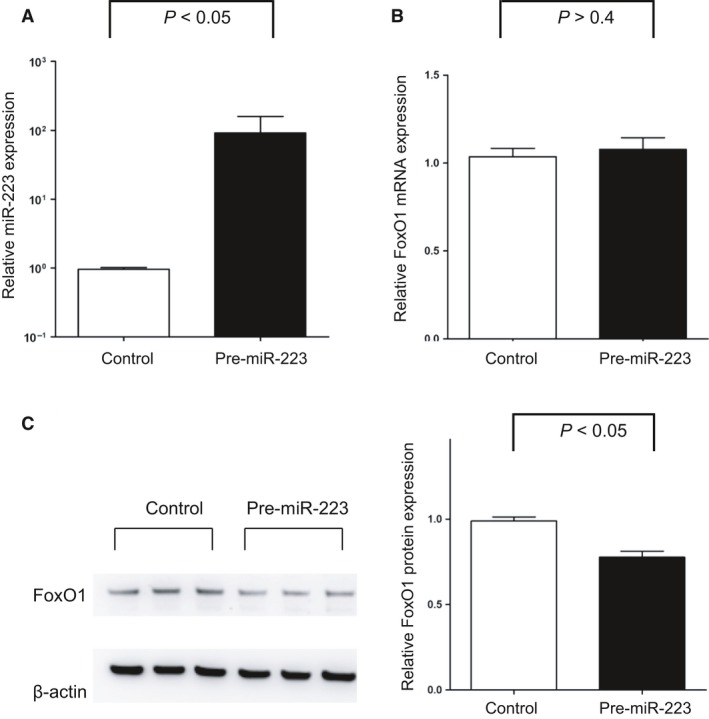
Promotion of FoxO1 mRNA by miR‐223 in Jurkat cells. (**A**) Transfection of pre‐miR‐223‐3p into Jurkat cells induced a 95.8‐fold increase in miR‐223‐3p expression (*P *<* *0.05). (**B**) FoxO1 mRNA expression did not differ after the transfection of pre‐miR‐223‐3p (*P *>* *0.4). (**C**) FoxO1 protein expression decreased by 21.4% with pre‐miR‐223‐3p transfection (*P *<* *0.05) by immunoblotting. FoxO1: Forkhead box O1. Relative expressions are presented as mean ± S.E.M.

As the foetal thymus showed a more prominent increase in miR‐223 expression in cases with acute chorioamnionitis, we carried out a computational search (www.targetscan.org: TargetScan release 6.2) to determine a functionally and clinically relevant target of miR‐223 in T cells and found that FoxO1, a member of the forkhead box O (FoxO) family of transcription factors, is among the putative targets of miR‐223. Therefore, Jurkat cells (Human T lymphocyte cell line) were used for the experiments to identify a putative target of miR‐223. Transfection of pre‐miR‐223‐3p into Jurkat cells induced a 95.8‐fold increase in miR‐223 expression (*P *<* *0.05, Fig. 2A). While FoxO1 mRNA expression did not differ after the transfection of pre‐miR‐223‐3p (*P *>* *0.4, Fig. 2B), FoxO1 protein expression decreased by 21.4% with pre‐miR‐223‐3p transfection (*P *<* *0.05, Fig. 2C) by immunoblotting.

**Figure 3:**
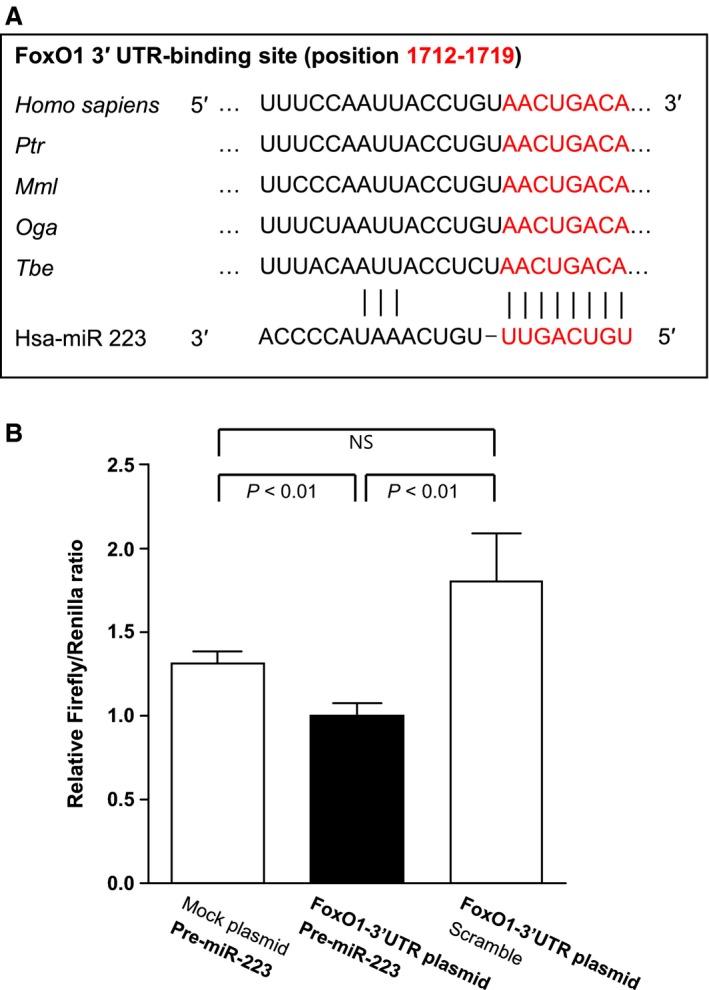
miR‐223 binding to the 3′ UTR of FoxO1 mRNA in luciferase assay. (**A**) The sequence in the 3′ UTR of FoxO1 mRNA (Human FoxO1 NM_002015 3′UTR, length: 3385) (positions 1712–1719) was predicted to bind miR‐223 and is conserved in several species (Ptr, Pan troglodytes; Mml, Macaca mulatta; Oga, Otolemur garnettii; Tbe, Tupaia belangeri). (**B**) Cotransfection with a reporter plasmid containing FoxO1 3′ UTR and pre‐miR‐223‐3p induced decreases in the luciferase activity compared to cotransfection with a mock reporter plasmid and pre‐miR‐223‐3p (27.1% decrease, *P *<* *0.01) or cotransfection with a reporter plasmid containing FoxO1 3′ UTR and miR‐223 precursor molecules‐negative controls (scramble) (44.6% decrease, *P *<* *0.01). FoxO1: Forkhead box O1, NS: not significant. Relative Firefly/Renilla ratios are presented as mean ± S.E.M.

**Figure 4:**
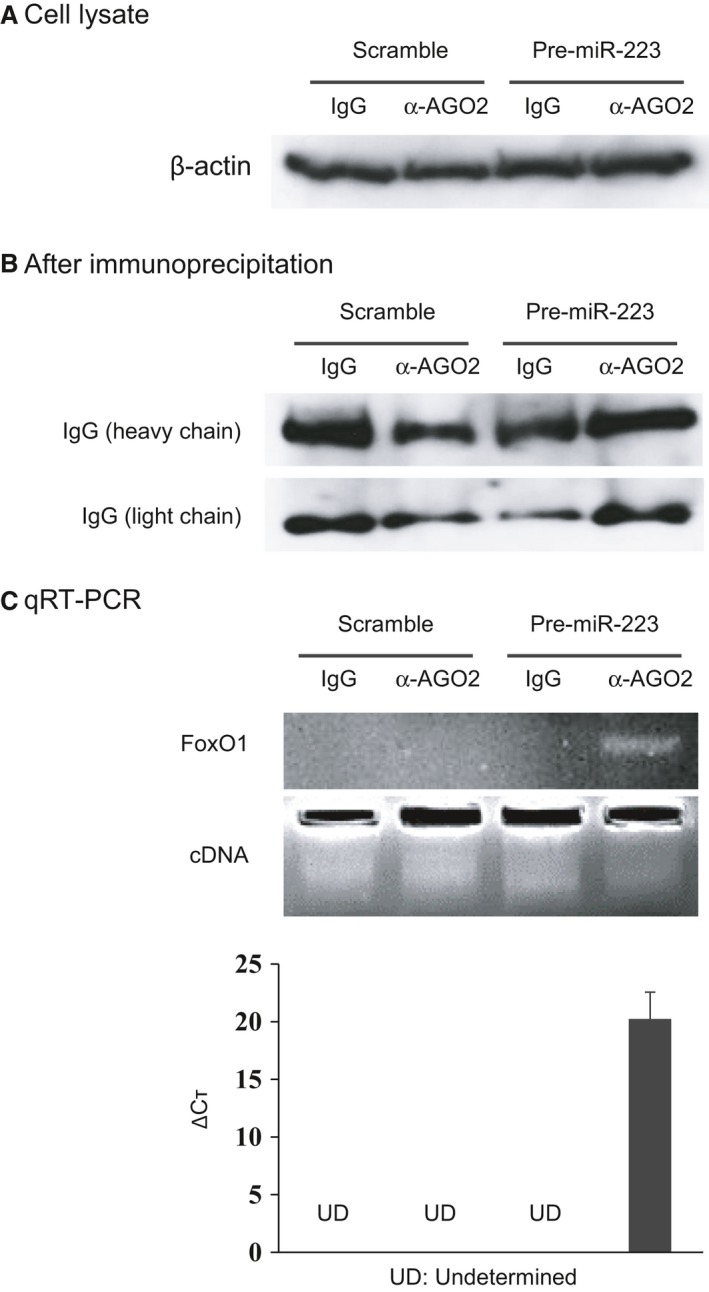
miR‐223 binding to the 3′ UTR of FoxO1 mRNA in ribonucleoprotein immunoprecipitation (RNP‐IP). (**A**) The expressions of β‐actin in the cell lysate were similar, irrespective of cotransfection with pre‐miR‐223‐3p (*versus* negative control: scramble) and anti‐argonaute 2 (AGO2) antibody (*versus* normal rat IgG antibody). (**B**) The amounts of heavy‐chain and light‐chain IgG in the beads after immunoprecipitation were similar, irrespective of cotransfection with pre‐miR‐223‐3p (*versus* negative control: scramble) and anti‐AGO2 antibody (*versus* normal rat IgG antibody). (**C**) FoxO1 mRNA was enriched only in the immunoprecipitates containing miR‐223‐loaded AGO2, whereas FoxO1 mRNA was not significantly enriched in the AGO2 IP sample in which the negative control molecule was transfected. AGO, argonaute‐2, UD, undetermined, FoxO1, Forkhead box O1.

To confirm the binding of miR‐223 to the 3′ UTR of FoxO1 mRNA, a transient transfection experiment was conducted using a luciferase reporter plasmid with the FoxO1 3′ UTR containing a putative miR‐223 binding site (Fig. 3). The sequence in the 3′ UTR of FoxO1 mRNA (Human FoxO1 NM_002015 3′ UTR length: 3385) (positions 1712‐1719) was predicted to bind miR‐223 and is conserved in several species, including Pan troglodytes (chimpanzee), Macaca mulatta (rhesus), Otolemur garnettii (bushbaby) and Tupaia belangeri (treeshrew). Cotransfection with a reporter plasmid containing the FoxO1 3′ UTR and pre‐miR‐223‐3p induced decreases in luciferase activity, compared to cotransfection with a mock reporter plasmid and pre‐miR‐223‐3p (27.1% decrease, *P *<* *0.01, Fig. 3B) or cotransfection with a reporter plasmid containing the FoxO1 3′ UTR and miR‐223 precursor molecules‐negative controls (scramble) (44.6% decrease, *P *<* *0.01, Fig. 3B). In addition to luciferase assay, ribonucleoprotein immunoprecipitation (RNP‐IP) followed by qRT‐PCR analysis was performed to demonstrate the physical interaction between miR‐223 and FoxO1 mRNA (Fig. 4). Jurkat cells were transfected with miR‐223‐3p precursor or negative control (scramble), and co‐immunoprecipitation of cell lysates was performed using anti‐argonaute 2 (AGO2) antibody. For the immunoprecipitates containing miR‐223‐loaded AGO2, which is a major component of the RNA‐induced silencing complex (RISC), the direct interaction between miR‐223 and FoxO1 mRNA was analysed by qRT‐PCR. As shown in Figure 4, FoxO1 mRNA was enriched only in the AGO2 IP sample in which miR‐223 was transfected, whereas there was no significant enrichment of FoxO1 mRNA in the AGO2 IP sample in which the negative control molecule (scramble) was transfected (delta Ct value: 20.24, Fig. 4C). Taken together, these data indicate that miR‐223 directly binds to the 3′ UTR of FoxO1 mRNA and negatively regulates the protein expression of FoxO1 mRNA through interaction with miR‐223‐loaded AGO2/RISC.

### FoxO1 protein expression in foetal thymus

**Figure 5:**
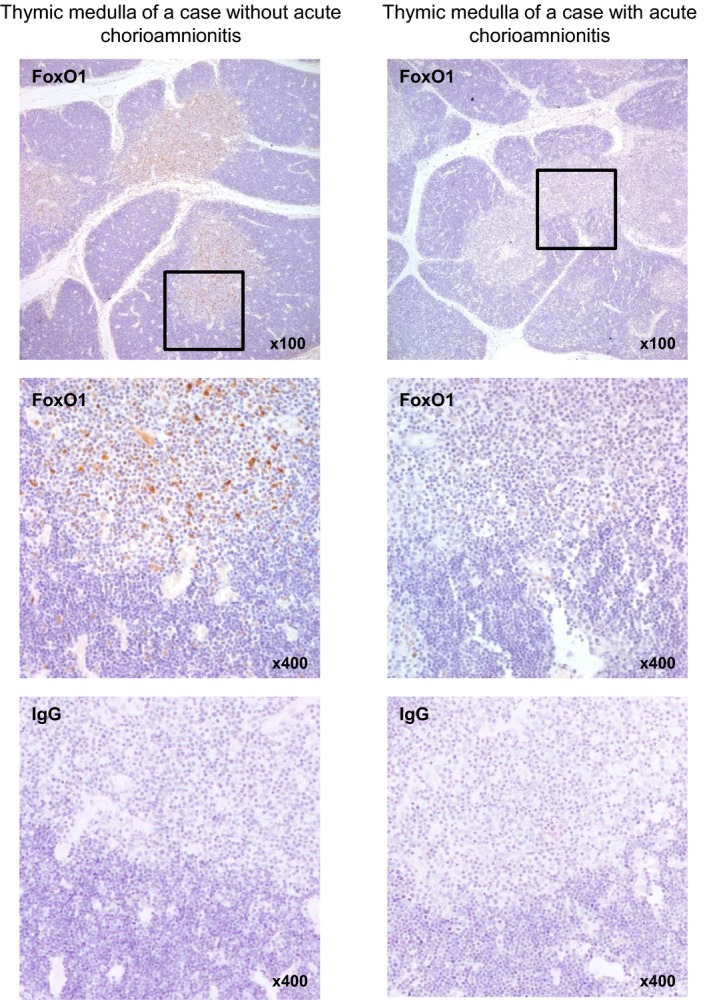
FoxO1 protein expression in foetal thymus. Immunohistochemistry images from a representative case show that cases with acute chorioamnionitis had lower FoxO1 protein expression in the thymic medulla than those without acute chorioamnionitis. Non‐specific binding was not observed in the IgG isotype controls (bottom). FoxO1, Forkhead box O1.

We further studied FoxO1 protein expression in the foetal thymus through immunohistochemistry (*N *=* *5 for control *versus N *=* *11 for acute chorioamnionitis, Fig. 5). When FoxO1 protein expression was assessed using a four‐tiered grading system (0, 1+, 2+, 3+), the cases with acute chorioamnionitis showed a significantly decreased FoxO1 immunoreactivity in the medullary thymus than did those without acute chorioamnionitis did (*P *=* *0.015).

## Discussion

The principal findings of this study are the following: (*i*) miR‐223 expression increases with acute chorioamnionitis in foetal thymus, lung and liver; (*ii*) FoxO1 is a target of miR‐223 in Jurkat cells; and (*iii*) FoxO1 protein immunoreactivity decreases in foetal thymus with acute chorioamnionitis.

MiRNAs are known to be new regulators of immune cell development and function 29, 30, and miR‐223 specifically has been reported to be involved in the differentiation and maturation of granulocytes 31, 32. The disruption of the physiological function of miR‐223 in granulocyte differentiation is associated with the development of acute leukaemia 33, 34. Because of its role in the regulation of the immune response, miR‐223 also has been proposed to be a potential prognostic and therapeutic target for the inflammatory disorders 35. In the current study, foetal thymus, lung and liver tissues were selected to determine the differential expression of miR‐223 in the context of acute chorioamnionitis, as those foetal organs are known to be associated with foetal immunity and inflammation. It is also well‐known that foetal thymus, liver and bone marrow are central to the development of the foetal immune system 36, 37. A large body of evidence indicates that acute placental inflammation is associated with inflammation in the foetal lung and is ultimately associated with foetal lung maturity 38, 39, 40. The expression pattern of miR‐223 in these foetal organs indicates that the biologic responses of the foetus to intra‐amniotic bacterial infection occur in multiple foetal organs, and this is consistent with the findings of previous studies that chorioamnionitis may lead to multi‐organ foetal diseases affecting the brain, lung, intestines and thymus 2, 41, 42, 43, 44, 45, 46, 47.

The observations in the present study provide the biological evidence that miR‐223 regulates FoxO1. FoxO1 is a member of the forkhead box O (FoxO) family of transcription factors that includes FoxO1, FoxO3, FoxO4 and FoxO6 48. FoxO1 plays an important role in the regulation of gluconeogenesis and glycogenolysis as well as a central role in the regulation of adipogenesis 49, 50. The FoxO transcription factor family also carries out significant roles in immune cell homeostasis and the regulation of inflammation in T cells, B cells and neutrophils 51, 52. These functions are strictly modulated by the ability of FoxO proteins to interact with various transcription factors in response to multiple external stimuli 53. When we performed a computational search (www.targetscan.org) to determine a functionally and clinically relevant target of miR‐223 in the context of acute chorioamnionitis, FoxO1 was among the putative targets of miR‐223.

The activity of FoxO transcription factors is known to be regulated by post‐translational modifications, such as phosphorylation, acetylation and ubiquitination 54. Recently, miRNA‐mediated post‐transcriptional modification has been reported as one of the regulation mechanisms that affect the activity of FoxO transcription factors. For example, miR‐182 55, miR‐155 56 and miR‐96 57 have an impact on various cellular functions by targeting FoxO3. In terms of FoxO1, Guttila and colleagues have demonstrated the regulation of FoxO1 by miR‐27a, miR‐96 and miR‐182 in breast cancer cells 58, and Hasseine *et al*. have studied the impact of miR‐139 on FoxO1 action by decreasing FoxO1 protein in mouse hepatocytes 59. Furthermore, Wu and colleagues have reported FoxO1 as the putative target of miR‐223 in colorectal cancer cells, cervical cancer cells and hepatoma cells 60. miRNAs regulate target genes through mRNA degradation/instability or the inhibition of translation of mRNAs 61, 62. The observations in the current study showed that miR‐223 regulates FoxO1 by decreasing FoxO1 at the protein level, but not at the mRNA level. This indicates that FoxO1 mRNA is silenced by miR‐223 mainly by translational repression, but not by FoxO1 mRNA decay.

The thymus is histologically divided into the cortex and medulla. The central cortex is the site of the early events in thymocyte development 63. The peripheral medulla, where the late events in thymocyte development take place, shows less dense cellularity but contains more mature T cells than the cortex does 63. The differential expression of FoxO1 protein according to the presence or absence of acute chorioamnionitis in the thymic medulla, but not in the thymic cortex, is intriguing. Based on the difference in the differential expression of FoxO1 between the two parts of the thymus, the possibility exists that miR‐223 and FoxO1 could affect the later stages of T‐cell development. A recent study on the changes in foetal thymus according to intrauterine inflammation reported a difference in terms of the foetal thymic immune cell populations of the cortex and medulla: the medulla was found to have a greater CD4:CD8 ratio than the cortex 64.

Several studies have investigated thymic changes in the context of chorioamnionitis. The association between foetal thymic involution and foetal inflammatory response syndrome has been previously reported 65, 66. Thymic changes after intrauterine inflammation, such as the decrease in thymus‐to‐body weight ratios 43 and alterations in foetal thymic immune cell populations 64, have also been reported. ‘Developmental programming’ during foetal or infantile periods is known to be closely associated with adult health problems, including coronary heart disease, stroke, hypertension and diabetes 67, 68. Thus, the long‐term consequences in the adaptive immune system and other organs by the alteration of miR‐223 and FoxO1 expression in the presence of placental inflammation need to be further clarified by additional observational and mechanistic studies. However, the findings in this study suggest that foetal programming by an abnormal intrauterine environment has potentially long‐term and systemic effects, as the alteration of miR‐223 occurs simultaneously in multiple organs and in the same direction. In addition, FoxO1 is a known regulator of gluconeogenesis, glycogenolysis and adipogenesis 49, as well as a modulator of the immune system and inflammation 50.

In summary, foetuses with inflammation in the chorioamniotic membranes showed increased expression of miR‐223 in the thymus, lung and liver, and FoxO1 is a target of miR‐223. These findings suggest that the post‐transcriptional regulation of genes by miR‐223 is a novel component of the foetal systemic inflammatory response to intrauterine infection and inflammation.

## Conflict of interest

The authors confirm that there are no conflict of interests.
